# Soft tissue infection caused by *Legionella bozemanii* in a patient with ongoing immunosuppressive treatment

**DOI:** 10.3402/iee.v3i0.20739

**Published:** 2013-09-06

**Authors:** Carl-Johan Neiderud, Angela Lagerqvist Vidh, Erik Salaneck

**Affiliations:** 1 Section of Infectious Diseases, Department of Medical Sciences, Uppsala University, Uppsala, Sweden; 2 Department of Clinical Microbiology, Uppsala University Hospital, Uppsala, Sweden

**Keywords:** Legionella bozemanii, soft tissue infection, immunosuppression, opportunistic infection

## Abstract

The *Legionellaceae* family consists of approximately 50 species, of which the most commonly identified species is *L. pneumophila*, the causative agent of Legionnaires’ disease. Other *Legionella ssp*. most often cause clinical infections in the immune-compromised patients, in which *L. bozemanii* has been known to cause both pneumonia and lung abscesses. In the presented case, a soft tissue infection in a patient with ongoing immunosuppression was determined to be due to *L. bozemanii*. Hence, in immune-deficient patients, *L. bozemanii* could be considered a possible agent in soft tissue infections when other common pathogens have been ruled out.

The Legionella bacteria are small Gram-negative rods with specific growth and culture requirements, which lead to certain diagnostic limitations. Approximately 50 species belonging to the *Legionellaceae* family have been identified, of which about 20 can cause infections in human beings. The most common clinical manifestation of these infections is pneumonia, and the vast majority of these are caused by *L. pneumophila*, commonly denominated Legionnaires’ disease. Mortality rates for Legionnaires’ disease vary widely, depending on the underlying condition of the patient as well as promptness of diagnosis and treatment. Legionella may also cause extra-pulmonary infections, such as Pontiac fever, which is almost always self-limited. The symptoms include flulike illness, with fever, headache, and myalgia (1). There are reports of skin and soft tissue infections caused by *Legionella spp*., including both *L. pneumophila* and other species, but these infections are rare and often appear in immunocompromised patients (2–9). Legionella species are found primarily in aqueous environments and in soil (10, 11), and thrive in artificial water and ventilation systems, which can disperse the bacteria causing outbreaks (12).

Although *L. pneumophila* can cause infection in immune competent subjects, other *Legionella* species, including *L. bozemanii*, are most frequently associated with some form of immune suppression, such as hematological malignancies, corticosteroid treatment, or other immune suppressive therapies (13). *L. bozemanii* has been described to cause pneumonia, both community acquired and nosocomial, whereas only a few case reports of lung abscesses have been published, all of these affecting immunosuppressed patients (14–16). We present here the first report of a soft tissue infection with *L. bozemanii*, in this case in an immunocompromised patient.

## Case

During a 3-year period, a previously healthy 82-year-old male repeatedly consulted a general practitioner as well as a rheumatologist because of joint pains. The patient was eventually diagnosed with seronegative rheumatoid arthritis. During this 3-year period, the patient was treated with increased doses of PO prednisolone to 20 mg daily and, eventually, the addition of methotrexate gradually increased to 15 mg weekly. Two series of intra-articular injections of methylprednisolone were administered to several metacarpophalangeal joints of the right hand as well as the right shoulder.

The patient was later admitted to the emergency department at the University Hospital, Uppsala, Sweden, 1 week after the last injections, with complaints of fever, chills, myalgia as well as an open wound on the right hand but denied any respiratory symptoms. The wound had appeared approximately at the time of the latest methylprednisolone injections and was open and purulent, with swelling of the dorsal side of the right hand. The left elbow and the dorsal side of the left hand were also swollen. The patient was circulatory stabile and afebrile. Auscultation of the heart and lungs revealed no abnormalities. Plasma CRP-levels (301 mg/l) and total leucocytes (15 × 10(9)/l) were elevated.

Blood and tissue samples were taken for routine cultures before treatment with cefotaxime 1 g IV q8h was started. Surgical exploration and debridement of the wound was performed upon arrival, revealing damage to extensor tendons of the forearm/hand. The swollen left elbow was also punctured and clear fluid was extracted and these particular symptoms were therefore suspected to be due to a non-infectious process, e.g. reactive arthritis. Several surgical revisions were performed during the first 10 days, due to the emergence of novel abscesses on the hands and forearms.

All cultures acquired prior to antibiotic therapy resulted negative. Due to non-satisfactory effect 11 days after admission, treatment was replaced with a combined therapy of imipenem + cilastatin (500 mg/500 mg IV q8h) and clindamycin (600 mg IV q8h). Supplementary cultures were taken, as well as biopsies subsequently analyzed for non-tuberculous mycobacteria (NTM) (culture, microscopy, and PCR), fungus (microscopy), Nocardia and Actinomyces (cultures). Samples were taken from wound material for bacterial 16S-RNA PCR. TBC-specific IGRA (QuantiFERON, Cellestis) turned out negative.

All further wound cultures as well as direct microscopy for acid-fast rods (*mycobacteria ssp*.) resulted negative. The 16S-RNA PCR did though indicated the presence of *L. bozemanii* in wound material. No reports of *L. bozemanii* causing soft tissue infections were previously reported and, therefore, NTM was still considered a possible cause and the therapy was now altered to target mycobacteria. A combination of moxifloxacin (400 mg PO q24h), amikacin (500 mg IV q24h), and clarithromycin (500 mg IV q12h) was introduced. Additional tissue samples were taken from the right upper arm and again 16S-RNA PCR was positive for *L. bozemanii*, and a Legionella-specific PCR was performed which also detected *L. bozemanii* DNA. Serological analysis was undertaken and compared to blood samples acquired on admission, which revealed a fivefold increase of anti-body titers of *L. bozemanii* and *L. longbeachae* serogroup 1 and 2 during a 12-day period. Treatment was now continued with moxifloxacin and clarithromycin.

Due to inadequate improvement of the patient's general condition a chest X-ray was performed on day 16. This revealed widespread perihilar consolidations, interpreted as alveolar edema. During the next 2 weeks, repeated X-rays showed only minor improvement, and a CT scan was therefore performed, revealing bilateral alveolar consolidations as well as consolidations in the left lung. These abnormalities were interpreted by radiologists to be due to a previous infection. During this period, several novel abscesses appeared on both upper limbs, requiring additional surgical evacuation.

However, the patient eventually did show clear general improvement as well as reduction of skin lesions and could be discharged from the hospital, after approximately 3 months. Treatment with moxifloxacin and clarithromycin was continued for a further 2 months. Follow-up examination was performed after a further 4 months. No new abscesses had developed and the previous lesions had completely healed.

## Discussion

This is the first reported case of *L. bozemanii* causing soft tissue infection. The patient was, at the time of presentation, undergoing treatment with both corticosteroids, per oral as well as with intra-articular injections, and methotrexate. Previously described manifestations of *L. bozemanii*, as well as other ‘non-pneumophila’ Legionella species, are primarily associated with ongoing immunosuppression, such as corticosteroid treatment. Extensive analyses, including PCR, cultures, and microscopy, were employed before serological methods could help conclude that the infecting agent was indeed *L. bozemanii*. Since this particular Legionella species had not previously been found to cause soft tissue infections, repeated analyses were required in order to definitely determine the diagnosis. The increase in antibody titers for *L. longbeachae* was interpreted as cross reactivity as several separate PCR analyses specifically and repeatedly identified *L. bozemanii* from wound tissue. In this case, these findings were considered relevant due to a majority of Legionella soft tissue infections being reported in patients with ongoing immune suppression (2–9).

No further epidemiological investigations were initiated and no plausible source of infection was identified. Other described cases of *L. bozemanii* infections have been found to have connection to aqueous exposure (15), as is the case with most Legionella species, including *L. pneumophila* (13). The patient's home was connected to the public water system, and no extensive exposure to water from private wells or bathing facilities could be identified. Gardening, one of the patient's recreational activities could also be considered as a mode of infection since soil is a reservoir for many Legionella bacteria (17). The radiological findings interpreted as a previous pulmonary infection could possibly indicate the respiratory tract as path of entry. Intra-articular steroid injections may subsequently have facilitated metastatic wound infections. In order to more clearly determine this scenario, further investigations of the respiratory tract would have be performed, such as bronchoscopy and bronchoalveolar lavage (BAL).

This case illustrates the need to consider Legionella species as a possible agent in soft tissue infections of immune-deficient patients. In the group of patients receiving immunosuppressive treatment, it may be likely that that this family of bacteria will be found to cause clinical manifestations that have so far not been reported. It is also likely that the difficulties in culturing Legionella bacteria have previously obscured diagnosis. In respect to this, further investigations of the manifestations of Legionella species during immunosuppressive treatment are highly motivated. In particular, *L. bozemanii* may be considered in immune-deficient patients with soft tissue infections.
